# Intermediate uveitis in Indian population

**DOI:** 10.1007/s12348-011-0020-3

**Published:** 2011-02-23

**Authors:** Swapnil Parchand, Manjari Tandan, Vishali Gupta, Amod Gupta

**Affiliations:** Department of Ophthalmology, Advanced Eye Centre, Post Graduate Institute of Medical Education and Research, Chandigarh, India

**Keywords:** Intermediate uveitis, Tuberculosis, Pars planitis, Sarcoidosis, Recurrences

## Abstract

**Purpose:**

Intermediate uveitis (IU) is generally believed to be autoimmune in nature requiring systemic corticosteroid and immunomodulatory therapy. This belief stems from the published reports from the developed countries; and the scenario maybe different in the developing countries that maybe endemic for certain infections. There are no large series available on the etiologic causes of intermediate uveitis from the developing countries. The present series aims to describe the etiology, treatment, and course of IU in North Indian population.

**Methods:**

In a retrospective analysis, records of 205 patients seen with a referral diagnosis of IU were retrieved and analyzed. After determining the etiology, 122 patients who had a definitive diagnosis of IU and a minimum follow-up of 1 year were analyzed further. All patients underwent investigations to rule out any possible etiology and received stepwise therapy comprising of depot or systemic corticosteroids, immunosuppressive/immunomodulatory therapy and pars plana vitrectomy. Specific therapy was administered wherever etiology could be determined. The primary outcome measure was recurrence of inflammation after a minimum of 6 months of initiating treatment.

**Results:**

There were 55 men and 67 women, and the disease was bilateral in 82 patients. Tuberculosis was the most common underlying etiology seen in 57 (46.7%), followed by sarcoidosis in 22 (18%), pars planitis in 35 (28.7%), and IU of idiopathic type in 8 (6.5%). Seventy three (59.8%) of 122 patients received systemic steroids, 55 (45.1%) were treated with periocular steroid, and 19 (15.6%) received immunomodulatory therapy. Specific antimicrobial therapy in the form of antitubercular treatment (ATT) was given in 42 patients. The recurrences were seen in 35 patients (28.7%) over a median follow-up of 18 months. Recurrences were seen more commonly in eyes with snow banking (*P* = 0.011); cystoid macular edema (*P* = 0.015), and in eyes that received local therapy (*P* = 0.001). Out of 57 patients who were diagnosed as intraocular tuberculosis, 42 patients (73.6%) received specific antitubercular treatment. Only 5 of 42 patients (11.9%) who received ATT had recurrence of inflammation compared to 7 out of 15 patients (46.7%) who did not receive ATT (*P* = 0.005).

**Conclusions:**

Tuberculosis is an important etiologic cause of IU in developing countries like India where the disease is endemic. It is important to investigate these patients as specific therapy with ATT helped in reducing the recurrences significantly.

Intermediate uveitis (IU) was defined by International Uveitis Study Group (IUSG) as idiopathic inflammatory syndrome, mainly involving the anterior vitreous, peripheral retina, and ciliary body with minimal or no anterior segment inflammation [1]. The term intermediate uveitis was used synonymously with pars planitis by IUSG and thus most of the intermediate uveitis was assumed to be idiopathic in etiology. The Standardization of Uveitis Nomenclature (SUN) Working Group defined IU as that subset of uveitis where vitreous is the major site of inflammation and the presence of peripheral vascular sheathing and macular edema should not change the classification [2]. The same group defined “pars planitis” as subset of IU where there is snowball or snow bank formation occurring in the absence of any systemic disease, i.e., idiopathic. [2] The etiology of IU is not well known and is mostly believed to be autoimmune, although it may be associated with systemic illness such as sarcoidosis, multiple sclerosis (MS) and several infectious diseases [3–6]. The disease is known for its prolonged course with exacerbations and hence the need for investigations to search for a specific etiology and proper management to reduce the recurrence and complications. The etiology may be variable in different parts of the world as it could be influenced by the geographic variations and ethnicity. The studies available from the developed countries [3–5] and one from North Africa [7] have mostly indicated IU to be of autoimmune in nature. In the cross-sectional epidemiologic studies done from the referral institutes in India, intermediate uveitis has been reported to be idiopathic in 77.5% in Northeast India [8], 91.4% in North India [9], and 81.6% in South India [10]. However, none of these studies describe the longitudinal course, management, and outcome of these patients labeled as intermediate uveitis. The present study was undertaken to find the etiologic spectrum, clinical manifestations, course, complications, and visual outcome in patients with IU from a single center in North India.

## Material and methods

We analyzed 15-year data (January 1995 through June 2009) from medical records of uveitis clinic of Post Graduate Institute of Medical Education and Research, Chandigarh, India. The medical record of all patients with diagnosis of IU as per the SUN working group criteria was analyzed in detail. Only patients with a minimum of 1-year follow-up were included. All the patients who did not complete a minimum 1 year of follow-up or where the disease showed significant granulomatous anterior segment inflammation during follow-up were excluded from further analysis. Patients of Fuchs uveitis and Behcet's disease who were initially misdiagnosed as IU were also excluded from further analysis.

All the enrolled patients had their records of history and ocular and systemic examination available at presentation as well as during follow-up visits. All patients had a detailed history recorded in the uveitis clinic file and underwent a complete ophthalmic examination including best corrected visual acuity, intraocular pressure, slit lamp biomicroscopy, and posterior segment examination with both slit lamp biomicroscopy and indirect ophthalmoscopy to examine pars plana area. Ancillary tests including fundus fluorescein angiography, optical coherence tomography, or ultrasound biomicroscopy, and specific laboratory tests were done as and when indicated. Systemic evaluation was done by a senior consultant internist who has special interest in systemic inflammatory diseases. Patients underwent baseline investigations including complete blood counts, erythrocyte sedimentation rate, tuberculin skin test, chest radiography, and *Treponema pallidum* hemagglutination test. Other targeted laboratory tests like serum angiotensin-converting enzyme level, Borrelia serology, chest computed tomography, magnetic resonance imaging (MRI) scan of the brain, and human leukocyte antigen typing were done whenever required. The following algorithm was followed: firstly, all the patients of Fuchs uveitis where vitritis was significant and mislead to the diagnosis of IU were excluded. Secondly, the patients with typical pars planitis as well as those with IU with/without snow banks and posterior synechia were investigated for infectious etiologies like tuberculosis (TB), sarcoidosis, Lyme and systemic associations like MS were ruled out. In patients with preponderant vitritis, etiologies like toxocariasis, lymphoma, candida, and Whipple's disease were ruled out. In patients with prepondrant vasculitis, MRI was done to rule out multiple sclerosis and intracranial lymphomas.

Patients were diagnosed as intermediate uveitis of presumed tubercular etiology if the following criteria were fulfilled: (1) a documented positive tuberculin skin test (10 mm of induration or more) at 48–72 h; (2) evidence of vitritis, snowballs, or snow banking; and (3) all known causes of infectious uveitis except TB and known noninfectious uveitic syndromes ruled out. Pars planitis was defined as subset of intermediate uveitis associated with snow bank or snowballs formation in the absence of an associated infection or systemic disease. When typical features of pars planitis are not present and the etiology could not be determined, then the term “intermediate uveitis of idiopathic type” was used.

The patients received treatment if (1) the visual acuity was worse than 20/40, (2) presence of cystoid macular edema, (3) vitreous haze of 2+ or more, and (4) retinal neovascularization. A stepwise graded approach to treatment included periocular steroid injection, systemic corticosteroids, immunosuppressive/immunomodular therapy, and pars plana vitrectomy. In cases with unilateral or asymmetric involvement, the periocular steroid injections were given first, and systemic treatment was initiated only in cases with insufficient effect and/or intolerance to this treatment modality. Systemic steroids (1–1.5 mg/kg body weight) were started in cases with severe bilateral disease and/or in cases with decrease visual acuity due to vitreous opacities. Immunosuppressive agents were started as a steroid-sparing drug or when steroid failed to control the inflammation. IU patients with presumed TB in addition also received antitubercular therapy (ATT). Pars plana vitrectomy was done if the vitritis was very severe at the time of presentation to our center despite receiving initial therapy outside and the laboratory investigations were equivocal and to manage the complications like retinal detachment and vitreous hemorrhage.

The primary outcome measure was the recurrence of inflammation occurring after a minimum of 6 months of receiving treatment. Visual improvement was defined as halving of the visual angle and visual deterioration as doubling of the visual angle. Visual acuity was said to be stabilized if the final visual acuity remained within two lines of the presenting acuity.

The statistical analysis was carried out using Statistical Package for Social Sciences (SPSS Inc., Chicago, IL, version 15.0 for Windows Evaluation Version). All quantitative variables were estimated using measures of central location (mean, median) and measures of dispersion (standard deviation). Normality of data was checked by measures of Kolmogorov–Smirnov tests of normality. For normally distributed data, means for more than two groups were compared using Kruskal–Wallis test. Qualitative or categorical variables were described as frequencies and proportions. Proportions were compared using chi-square test. All statistical tests were two sided and performed at a significance level of *α* = 0.05.

## Results

Our study included 205 patients with a diagnosis of IU seen between years 1996 and 2009. Of these 205 patients, 122 fulfilled the inclusion criteria with at least 1 year of follow-up and were analyzed further. There were 55 men (45.1%) and 67 women (54.9%) with male-to-female sex ratio of 1:1.2. The mean age at diagnosis was 36.1 ± 13.3 years (range 5–68 years). The disease was bilateral in 82 patients (67.2%). Presenting complaints were decreased vision in 100 patients (81.9%), redness in 52 patients (42.6%), pain in 42 patients (34.4%), and floaters in 40 patients (32.7%). The most common sign at presentation was vitritis seen in all 204 eyes (100%) and snowballs in 177 eyes (86.8%). Snow banking was present in 40 eyes (19.6%). Associated anterior segment inflammation was seen in 128 eyes (62.7%).

Of these 122 patients, 57 (46.7%) had underlying etiology of presumed TB and 22 patients (18%) had sarcoidosis. Thirty-five patients (28.7%) were diagnosed as pars planitis while 8 patients (6.5%) were diagnosed as IU of idiopathic type (Table 1). The clinical features at presentation were comparable in all the groups irrespective of etiology (Table 2).

**Table 1:** Demographic profiles of patients

Factors	IU of idiopathic type (*n* = 8 patients)	Presumed IOTB (*n* = 57 patients)	Pars planitis (*n* = 35 patients)	Sarcoidosis (*n* = 22 patients)	Overall (*N* = 122 patients)
Age	41 ± 12.8	37.5 ± 12.1	28.3 ± 13.4	42.2 ± 12.2	36.1 ± 13.3
Sex (M/F)	1:1.6	1:1.1	1.3:1	1:2.6	1:1.2
Bilateral	7 (87.5%)	37 (64.9%)	21 (60%)	17 (77.3%)	82 (67.2%)

*M* male, *F* female, *IU* intermediate uveitis

**Table 2:** Clinical signs at presentation

Factors	IU of idiopathic type (*n* = 14 eyes)	Presumed IOTB (*n* = 95 eyes)	Pars planitis (*n* = 56 eyes)	Sarcoidosis (*n* = 39 eyes)	Overall (*n* = 204 eyes)	*P* value
AC reaction	12 (85.7%)	61 (64.2%)	28 (50%)	27 (69.2%)	128 (62.7%)	0.165
Vitreous cells	14 (100%)	95 (100%)	56 (100%)	39 (100%)	204 (100%)	
Snowballs	10 (71.4%)	82 (86.3%)	49 (87.5%)	36 (92.3%)	177 (86.8%)	0.266
Snow banking	6 (42.9%)	16 (16.8%)	14 (25%)	4 (10.3%)	40 (19.6%)	0.037

*AC* anterior chamber, *IOTB* intraocular tuberculosis

Seventy three (59.8%) of 122 patients received systemic steroids, 55 patients (45.1%) were treated with periocular steroid. Nineteen patients (15.6%) received immunomodulatory therapy. Twelve patients (9.8%) with good visual acuity and minimal inflammation received only topical steroids. Specific antimicrobial therapy in the form of ATT was given in 42 patients.

The most common complications seen over the follow-up were the development of cystoid macular edema (CME) in 92 eyes (45.1%), cataract in 70 eyes (34.3%), glaucoma/ocular hypertension in 23 eyes (11.2%), and epiretinal membrane (ERM) in 29 eyes (14.2%). Other complications noted were optic disc pallor in nine eyes (4.4%), peripheral neovascularization in eight eyes (3.9%), retinoschisis in four eyes (1.9%), retinal detachment in two eyes (0.9%) and vitreous hemorrhage in one eye (0.5%) (Table 3). Of the eight patients developing retinal neovascularization, six had presumed intraocular tuberculosis (IOTB) as an underlying etiology. Thirty-five eyes (17.1%) underwent surgery for the complication of intermediate uveitis including cataract surgery with posterior intraocular lens implantation in 28 eyes (13.7%), three-port pars plana vitrectomy in 5 eyes (2.4%), and glaucoma filtering surgery in 2 eyes (0.9%). Nine patients underwent laser photocoagulation for peripheral neovascularization (six patients; 8 eyes), retinal breaks (two patients; 2 eyes), and for macroanuerysm (one patient; 1 eye).

**Table 3:** Complications associated with IU

Factors	IU of idiopathic type (*n* = 14 eyes)	Presumed IOTB (*n* = 95 eyes)	Pars planitis (*n* = 56 eyes)	Sarcoidosis (*n* = 39 eyes)	Overall (*n* = 204 eyes)
CME	9 (64.3%)	38 (40%)	25 (44.6%)	20 (51.3%)	92 (45.1%)
Cataract	8 (57.1%)	37 (38.9%)	7 (12.5%)	18 (46.2%)	70 (34.3%)
Glaucoma/ocular hypertension	1 (7.1%)	6 (6.3%)	11 (19.6%)	5 (12.8%)	23 (11.2%)
ERM	7 (50%)	9 (9.5%)	5 (8.9%)	8 (20.5%)	29 (14.2%)
Optic disc pallor	0	5 (5.3%)	2 (3.6%)	2 (5.1%)	9 (4.4%)
Peripheral neovascularization	0	5 (5.3%)	1 (1.8%)	2 (5.1%)	8 (3.9%)
Retinoschisis	0	2 (2.1%)	1 (1.7%)	1 (2.5%)	4 (1.9%)
Retinal detachment	0	1 (1.05%)	0	1 (2.5%)	2 (0.9%)
Vitreous hemorrhage	0	1 (1.05%)	0	0	1 (0.5%)

**Fig. 1:**
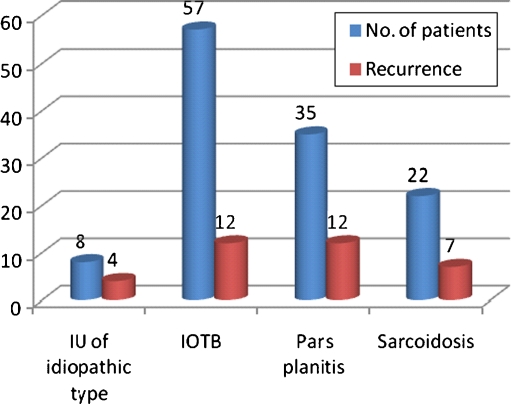
Recurrences in different etiologies. *IU* intermediate uveitis, *IOTB* intraocular tuberculosis

**Fig. 2:**
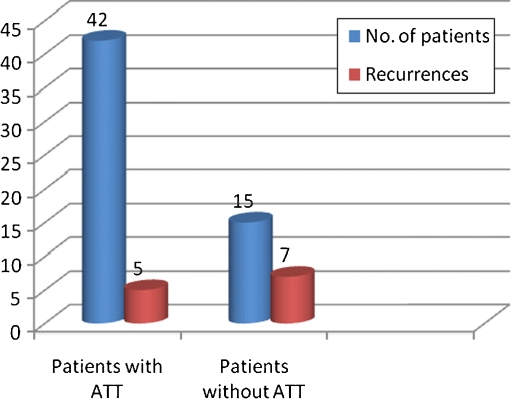
Comparison of recurrences within IU with presumed TB group. *ATT* antitubercular treatment

The recurrences were seen 35 patients (50 eyes) over follow-up ranging between 1 and 10 years (mean, 31.9 ± 30.21 months/median, 18 months) (Fig. 1). Recurrences were seen more commonly in eyes with snow banking with 16 of 40 eyes (40%) with snow banking having recurrence compared to 34 of 164 eyes (20.7%) without snow banking (*P* = 0.011). Recurrences were significantly higher in patients having cystoid macular edema with 30 of 92 eyes (32.6%) with CME having recurrence compared to 20 of 112 eyes without CME (17.9%) (*P* = 0.015). Patients receiving local therapy had higher recurrences compared to those receiving systemic therapy with 32 of 86 eyes (37.2%) receiving local therapy in the form of posterior sub -tenon triamcinolone had recurrence as compared to 18 of 118 eyes (15.2%) who did not received local therapy (*P* = 0.001). Out of 57 patients who were diagnosed as intraocular tuberculosis, 42 patients (73.6%) received specific antitubercular treatment. Eleven patients (19.2%) received only oral steroids without ATT and 4 patients received only topical steroids. Five of 42 patients (11.9%) who received ATT had recurrence of inflammation as compared to 7 out of 15 patients (46.7%) who did not receive ATT. The effect of antitubercular therapy in reducing the number of recurrences of IU in patients receiving ATT was found to be statistically significant (*P* = 0.005) (Figs. 1 and 2).

The mean visual acuity at presentation was 20/50 (range 20/20—counting fingers at 2 ft) and final visual acuity was 20/30 (range 20/20–20/400). After treatment, visual acuity improved in 107 eyes (52.4%), deteriorated in 16 eyes (7.8%), and stabilized in 81 eyes (39.7%). A final visual acuity of 20/40 or more could be achieved in 85 patients (69.6%). Factors associated with poor visual outcome were cataract (*P* = 0.0001), CME (*P* = 0.0001) and ERM (*P* = 0.01). There was no difference in the final visual outcome between different subgroups.

## Discussion

The overall age of presentation, gender distribution, and laterality were comparable to previously reported series [3–5, 11–13]. Vitritis was the most common finding seen in our series seen in all the patients and snow banking was seen in 20% of eyes; a finding that is in accordance with the previously reported series. However, the number of eyes showing snowballs at presentation was significantly higher in our series compared to previously published series [7]. This could be due to the presence of TB as an etiology cause in 46.7% of our patients as the eyes with TB had higher incidence of snowballs seen at the time of presentation compared to those with non-tubercular etiologies.

In the present series including patients from India, a country endemic for tuberculosis, nearly 46.7% cases with IU had TB as an etiology. This is a significant finding as none of the previous series from other parts of the world have reported TB as an important underlying cause of IU [3–6]. On the contrary, the study from Northeast India did report TB and sarcoidosis as important etiologies in IU [8]. In our previous report, we had analyzed the uveitis patients seen between years 1996 and 2001 and reported TB and sarcoidosis seen in 4% each in cases with IU [9]. However, the different set of criteria was used for making a diagnosis of tubercular uveitis [9]. In the previously published data [9], patient was diagnosed as presumed IOTB if he/she fulfilled any two of the three criteria including (a) clinical suspicion of the disease where any two of the following features were present: (1) granulomatous anterior uveitis, (2) active periphlebitis, (3) neuroretinitis, (4) retinochoroiditis, and. (5) subretinal abscess/granuloma; (b) corroborative evidence of disease: (1) Mantoux >20 mm/necrosis, (2) positive chest X-ray, (3) aqueous or vitreous tap positive for *Mycobacterium tuberculosis* (PCR), (4) sputum positive for acid-fast bacilli on smear, culture, or both, and (5) histopathological evidence of tuberculosis from cervical or parahilar lymph nodes; and (c) response to ATT. Since the presence of vitritis alone did not qualify the clinical criteria for presumed IOTB, many of these patients were not diagnosed as TB. In the current study, the inclusion criteria were less stringent and modified to include the evidence of vitritis, snowballs, or snow banking as clinical features with a documented positive tuberculin skin test (10 mm of induration or more).

It is important to emphasize this issue because many ophthalmologists may not routinely investigate these patients for TB which can lead to prolonged disease course with multiple recurrences for want of receiving specific antituberculosis therapy. The administration of ATT led to significant reduction in the number of recurrences during follow-up in the present series, thus highlighting the benefit of adding timely ATT in these patients. There were no clinical indicators predictive of possible tubercular etiology at presentation but during follow-up, the eyes with presumed tubercular IU had more tendency to develop retinal neovascularization. Thus, it is important to keep these eyes under close monitoring for the development of areas of capillary nonperfusion and retinal neovascularization.

The visual prognosis was good in present series as about 70% patients could achieve a final visual acuity of 20/40 or better. Presence of cystoid macular edema, cataract, and epiretinal membranes were associated with poor visual outcome. Since all these factors indicate an irreversible cause of visual loss, many of these are likely to improve further after the surgical management, thus improving the prognosis even further.

In summary, the results of present series indicate that in Indian population, IU is more likely to be due to TB than multiple sclerosis or any other etiology. Timely management of these patients leads to improvement/stabilization of vision in 93% of the eye with 70% achieving a final visual acuity of 20/40 or better.
